# Is the oral pathogen, *Porphyromona gingivalis*, associated to colorectal cancer?: a systematic review

**DOI:** 10.1186/s12885-025-13770-4

**Published:** 2025-03-04

**Authors:** Adrián Navarro-Sánchez, María Ángeles Nieto-Vitoria, José Antonio López-López, Juan José Martínez-Crespo, Fernando Navarro-Mateu

**Affiliations:** 1 University of Murcia, Murcia, Spain; 2 Digestive Service, General University Hospital Reina Sofía, Murcia, Spain; 3 Department of Methodology and Basic Psychology, Meta-Analysis Unit, University of Murcia, Murcia, Spain; 4 Research Institute IMIB-Pascual Parrilla, Murcia, Spain; 5 Mental Health Research and Training Unit, Murcian Health Service, Murcia, Spain; 6 CIBER de Epidemiología y Salud Pública (CIBERESP), Madrid, Spain

**Keywords:** Colorectal cancer, CRC, Porphyromonas, Porphyromonas gingivalis, Microbiota

## Abstract

**Background:**

The association between the oral pathogen *Porphyromonas gingivalis* (PG) and the gut microbiota in colorectal cancer (CRC) patients has been explored with inconsistent results. This study aims to systematically assess this potential association.

**Materials and methods:**

A systematic review was conducted across three databases (Pubmed, Embase and Web of Science) from inception up to January 2023 and updated until November 2024. Inclusion criteria were observational studies examining PG in the microbiota of adults with CRC compared to healthy controls. Exclusion criteria were studies without control group of healthy individuals, other designs or without full-text access. Two reviewers independently selected and extracted data following a pre-registered protocol. Disagreements were resolved by consensus or with a third reviewer. Risk of bias (RoB) was assessed using the Newcastle–Ottawa Scale (NOS). Results were summarized with a flow diagram, tables, and narrative descriptions. Meta-analysis was not feasible, so Fisher’s method for combining p-values and the sign test were used as alternative integration methods.

**Results:**

Finally, 18 studies, with 23 analysis units were included, providing a total sample of 4,373 participants (48.0% cases and 52.0%controls), 38.2% men and 61.8% women, with a similar distribution among cases and controls. The mean (SD) age of cases was 63.3 (4.382) years old and 57.0 (7.753) years for controls. Most of the studies analyzed the presence of PG in feces (70.0%) collected before colonoscopy (55.0%) and were classified with good quality (70.0%) in the RoB assessment. Results suggested an effect (Fisher’s test, p = .000006) with some evidence towards a positive association of PG in CRC patients compared to healthy controls (Sign test, *p* = .039).

**Conclusions:**

Results of the systematic review suggest that PG is associated with the microbiota of CRC patients. Lack of information to calculate the effect size prevented the performance of a meta-analysis. Future research should aim for standardized protocols and statistical approaches.

**Funding:**

No funding was received for this work.

**Systematic review registration:**

The research protocol was registered with the International Prospective Register of Systematic Reviews (PROSPERO) on 2023 (registration number: CRD42023399382).

**Supplementary Information:**

The online version contains supplementary material available at 10.1186/s12885-025-13770-4.

## Introduction

Colorectal cancer (CRC) is the third most common and the second most lethal cancer globally [1], with 1.9 million incidence cases and 0.9 million deaths reported worldwide in 2020 [2]. CRC is considered an etiologically heterogeneous disease influenced by both genetic factors and modifiable environmental risks, including smoking, unhealthy diet, excessive alcohol consumption, physical inactivity, and obesity [1, 3]. Dysbiosis, or disruption of the healthy gut microbiota, has been associated to CRC, as highlighted by several systematic reviews and meta-analyses [4–9]. Gut microbiota imbalances have also been associated with other cancers and a wide range of health outcomes, including immune, metabolic, neurological, and mental health conditions [10–19]. To better understand this complex relationship, it is essential to identify key microbiota components involved in the carcinogenic process [20].

Periodontitis is a chronic inflammation of the periodontium, leading to tooth loss due to progressive destruction of the supporting tissues dental support tissue and alveolar bone [21, 22]. Primarily caused by oral bacterial infection, periodontitis has been linked to various systemic conditions, including cancer, and diseases affecting the gastrointestinal, cardiovascular, endocrine, respiratory, and central nervous system [22–25]. Two key pathogens involved in periodontitis, *Fusobacterium nucleatum* (FN) and *Porphyromonas gingivalis* (PG), have shown associations with CRC [26, 27]. While several systematic reviews and meta-analyses have examined the role of FN in CRC development [28–32], the specific and independent role of PG has not yet been systematically assessed.

PG, a Gram-negative anaerobic bacterium, is considered a major etiological agent in severe periodontitis [33, 34] and is found in nearly 86% of subgingival plaque samples [35]. Notably, PG has been associated with both cancer incidence and prognosis [25], and specifically with CRC [36*, 37*, 38*]. However, this association remains controversial due to conflicting results. While some studies have reported higher concentrations of PG in CRC cases compared to healthy controls [36*, 37*, 38*, 39*, 40*], others have shown the opposite trend [41*, 42*, 43*] or found no association [44*].

The aim of this study was to systematically review the association of PG and CRC and to identify potential risks of bias in scientific literature. The research question was formulated using the PECO format (Population, Exposure, Comparator, and Outcomes) [45] as: do people (P) with CRC (O) have a different concentration of PG (E) compared to healthy controls (C)?

## Materials and methods

This systematic review is reported according to the PRISMA 2020 guidelines (Preferred Reporting Items for Systematic Reviews and Meta-Analyses) [46] in conjunction with the SWIM guidelines (Synthesis without Meta-analysis) [47]. It was previously registered in the International Prospective Register of Systematic Reviews (PROSPERO 2023 registration code: CRD42023399382 (available at www.crd.york.ac.uk/prospero/)).

### Study eligibility criteria

Inclusion criteria according to the PECO strategy were as follows: a) Population: adult population; b) Exposure: studies focused on the analysis of PG in the microbiome of participants; c) Comparison: a healthy control group without CRC or precancerous lesions; d) Outcome: a diagnosis of CRC; and e) Study designs: observational studies (case–control, cohort or cross-sectional) published in English and/or Spanish. Exclusion criteria were a) Studies with no control group of healthy individuals without benign colon pathology (e.g., adenomatous polyps); b) Other study designs (e.g., reviews, case-only or case series, non-human studies, communications to conferences or posters, letters to the editor or opinion papers); c) Studies reported in a format other than journal article (e.g., contributions to conferences, letters to the editor, theses, or opinion papers); and d) Studies without access to the full text. A list of excluded studies with their reason for exclusion was elaborated.

### Information sources and search strategy

Comprehensive electronic searches were conducted to identify studies indexed in three databases: PubMed/MEDLINE, EMBASE and Web of Science from inception until January 2023. This first systematic search was updated up on November 2024 to reduce the time period between search and publication to less than 2 years, following the AMSTAR 2 quality criteria [48]. The following search terms were used in both search strategies: (colorectal cancer OR colon cancer OR rectal cancer OR cancer of the colorectum OR cancer of the colon OR cancer of the rectum OR colorectal neoplasm OR colon neoplasm OR rectal neoplasm OR colorectal tumour OR colon tumour OR rectal tumour OR colorectal carcinoma OR colon carcinoma OR rectal carcinoma) AND (*Porphyromonas* OR *Porphyromonas Gingivalis*) (see Supplementary Table 1 for a specific description of the search strategy used in each of the databases). No filters or restrictions related to time frame, sample size, ethnicity/race, type of sample, extraction method, or microbiota analysis were placed in the search strategy. The references cited in each included study and the review articles found were manually searched to identify other potentially eligible studies. To describe the study selection process, a flow chart was used following the PRISMA 2020 guidelines [46].

### Selection process and data extraction

The titles and abstracts of initial studies identified were manually screened to remove duplicates and clearly ineligible studies. Next, full-text articles were evaluated according to the inclusion and exclusion criteria. For included studies, we extracted data on the following categories using a previously defined protocol: i) identification data of the study (e.g., author, country, journal, language and year of publication); ii) methods (e.g., study design, sample sizes, case definition, and variables adjusted for in the statistical analyses); iii) sample characteristics of cases and controls independently (e.g., percentage of males, mean age, BMI (Body Mass Index) and ethnicity, among others); iv) risk of bias assessment (described in more detail below); v) information related to the CRC cases (e.g., diagnostic confirmation of the CRC, exclusion from the study due to any gastrointestinal illness such as inflammatory bowel disease or any restriction of last antibiotic consumption, among others); vi) exposure definition related to PG (e.g., type of biological sample (biopsy, feces or saliva samples) and time of sample collection, frequency of PG in specimens, technique used to detect and quantify the bacterium load, and main results, among others). If an included article reported two or more studies with independent samples, or the analyses were performed on different tissues from the same participants, they were considered as independent units of analysis in the database. Two reviewers (ANS and MAN) independently participated in both the selection and the data extraction processes. Disagreements were resolved by consensus or by the intervention of a third reviewer (FNM).

### Risk of bias assessment

The risk of bias of each of the selected studies was assessed using the Newcastle–Ottawa Scale (NOS) for observational studies [49]. The NOS consists of eight items grouped into three dimensions: selection, comparability, and exposure. A star scoring system is used to evaluate the quality. A total quality score (TQS) of each individual study was calculated by adding all stars (range = 0–9, with a higher score indicating higher overall quality). Study quality was categorized as good, fair, or poor, according to the Agency for Healthcare Research and Quality (AHRQ) thresholds for converting NOS scores) [17, 30, 50]: i) Good quality: 3 or 4 stars in selection domain AND 1 or 2 stars in comparability domain AND 2 or 3 stars in outcome/exposure domain; ii) Fair quality: 2 stars in selection domain AND 1 or 2 stars in comparability domain AND 2 or 3stars in outcome/exposure domain; iii) Poor quality: 0 or 1 star in selection domain OR 0 stars in comparability domain OR 0 or 1 stars in outcome/exposure domain. In addition, two other characteristics of the studies that could be related to a potential risk of bias specifically related to the analysis of the samples were added: whether it was specified that analyses were performed blind to the case/control status and whether any quality assessment procedures were implemented in the measurement of PG. A tabulated synthesis and a narrative description of results of included studies were carried out, as well as a description of the risk of bias assessment of individual included studies. The Grading of Recommendations Assessment, Development and Evaluation (GRADE) approach was used to globally evaluate the quality of evidence [51] and presented using the Cochrane’s template for assessing the GRADE criteria [52].

### Statistical analysis and synthesis of results

To evaluate the reliability of the selection and data extraction processes, the inter-rater agreement was calculated using Cohen's kappa coefficient. Although the protocol for this systematic review stated that meta-analysis would be used for the statistical integration of findings, this was not feasible due to the lack of information in the primary studies to compute an effect measure. Instead, alternative synthesis methods were considered following guidelines from the Cochrane Handbook [53]. First, we undertook a combination of p-values using the method proposed by Fisher [54] to address the question ‘*Is there evidence of an association between PG and CRC in at least one study?*’ Second, we used vote counting and performed a sign test to answer the question ‘*Is there any evidence of a higher PG concentration in the gut microbiota of CRC patients compared to healthy controls?*’. As no meta-analysis was performed, it was not possible to properly assess heterogeneity. Fisher’s exact test was used to evaluate differences in different variables between those analysis units with and without an increase in PG concentration among CRC patients. Variables analyzed included reporting BMI of participants, exclusion criteria of participants (antecedents of digestive tract diseases, prior antibiotic use, use of probiotics or prebiotics), sample type and when they were collected, quantification measures reported, and the two NOS items with less than 75% of adherence (NOS-S.3 Representativeness of cases; and NOS-E.3 Non-response rate reported). For both tests, we considered the standard 5% threshold for statistical significance (*p*-value ≤ 0.05). All statistical analyses considered an alpha level of 0.5 and were conducted in R [55].

## Results

### Study eligibility and data extraction

**Fig. 1:**
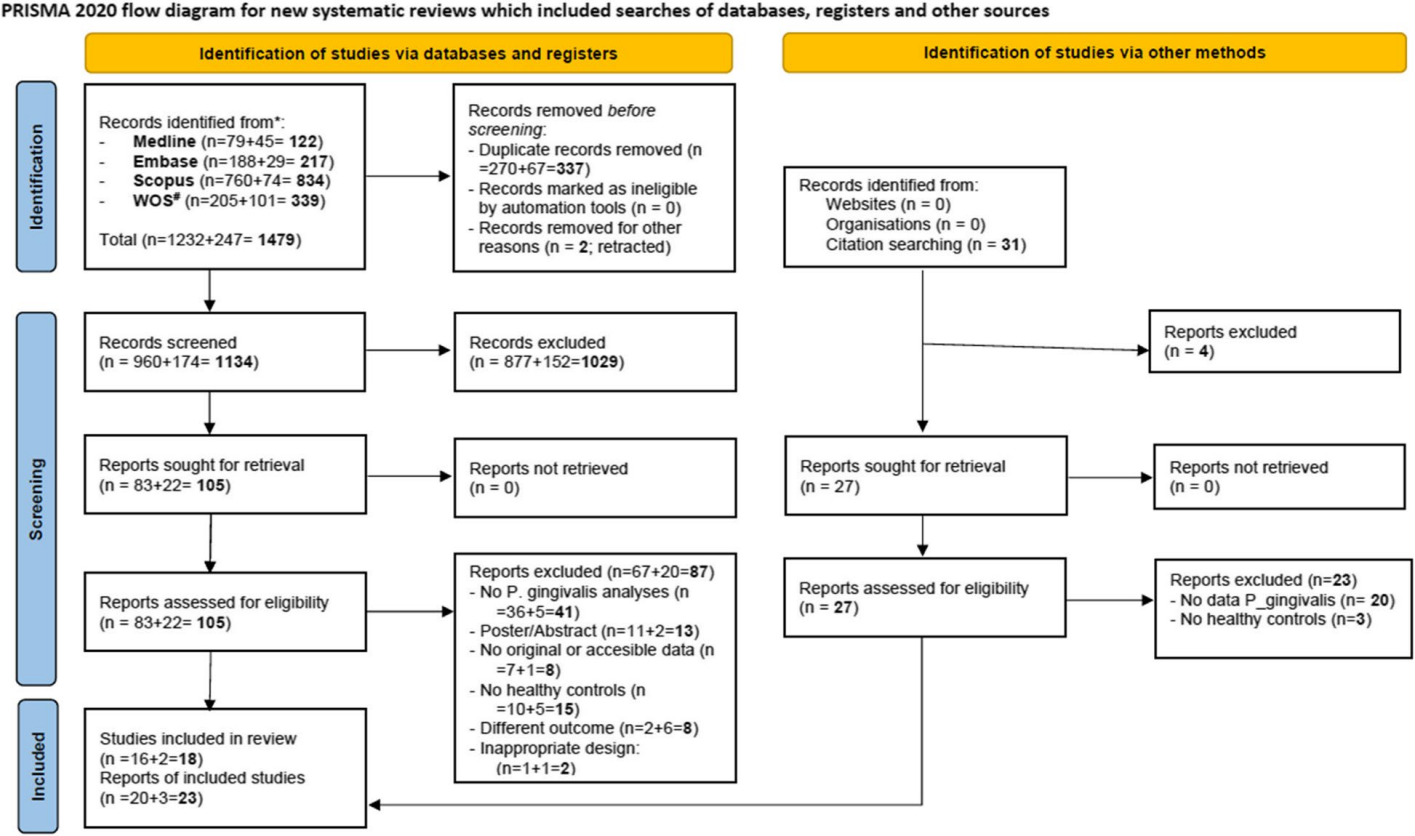
Flow diagram describing the search and selection process

Figure 1 provides an overview of the two systematic searches and selection processes using a flow diagram. A total of 1,479 articles were initially found with 31 additional studies identified through backward searching of references from the included studies. After removing 337 duplicates and two retracted articles, 1,134 remained for screening. A preliminary review of titles and abstracts led to the exclusion of 1,029 articles. The full texts of the remaining 105 articles were evaluated against the inclusion and exclusion criteria, resulting in the exclusion of 87 articles (see Supplementary Table 2 for the list of excluded studies and the primary reasons for exclusion). Finally, 18 studies were included in the systematic review [36*, 37*, 38*, 39*, 40*, 41*, 42*, 43*, 44*, 56*, 57*, 58*, 59*, 60*, 61*, 62*, 63*, 64*]. Four of the included articles published results from different cohorts [57*, 58*, 61*, 64*], leading to a total of 23 analysis units across 18 studies. The mean (SD) Cohen's kappa inter-rater agreement coefficient was 0.59 (0.26) for the selection process and 0.68 (0.32) for data extraction.

### General description of included articles

A total of 23 units of analysis from 18 observational studies were included in the systematic review, encompassing 4,373 participants (2,098 (48%) cases and 2,275 (52.0%) controls). The general characteristics and main results of the included articles are summarized in Table 1. Sample sizes varied significantly, ranging from 17 participants [41*] to 764 [59*]. Sex distribution of the total sample included 1,672 (38.2%) men and 2,703 women (61.8%) with a similar distribution within cases (786 (37.5%) men and 1,312 (62.5%) women) and controls (886 (38.9%) men and 1391 (61.1%) women). The mean (SD) age of the cases was 63.3 (4.392) years, compared to 57.0 (7.753) years for controls. Ethnicity was considered in three articles (13.0%), which primarily distinguished between African-American participants, Caucasians, and other ethnicities.

**Table 1:** Description of included studies

					**Exclusion criteria**				
**Author, year**	**Country**	**Nº Case/** **Control**	**Nº men Case/** **Control**	**Race Case/Control (Nº (%))**	**History of digestive tract diseases**	**History of antibiotic use prior to sample** (time period)	**Other exclusion criteria**	**Biologic samples**	**Sample collection time**
Adel El-Sokkary 2022 [39]	Egypt	27 / 7	12 / -	-	No	No	-	Feces	-
Conde-Pérez 2024 [56*]	Spain	93 / 30	57 / 7	-	Yes	Yes (1 month)	Infectious (< 3 months) or immunological diseases, Chemotherapy/radiotherapy, Genomic predisposition to CRC, Transplants/ immunosuppresor treatments	Feces, saliva and GCF^##^ samples	Before colonoscopy
Fukugaiti 2015 [41*]	Brasil	7 / 10	5 / 8	-	Yes	Yes	Any systemic infection	Feces	Before colonoscopy
Gao 2020 [40*]	China	100 / 332	61 / 232	-	Yes	Yes (last month)	Cancer patients: underwent radiology or chemotherapy Healthy controls: any cancer history, administered proton pump inhibitor, probiotics, prebiotics or synbiotics within 1 month, had underwent surgery on digestive tract	Feces	After colonoscopy
Gao 2021 [36*]	China	71 / 91	42 / 38	-	Yes	Yes (last month)	Gastrointestinal surgery, probiotics of prebiotic consumption within 1 moth,	Feces	Before (Cases: 43; Controls:40) After (Cases:28; Controls:51)
Guven 2019 [42*]	Turkey	71 / 77	46 / 37		Yes	Yes	Hereditary CRC syndromes	Saliva	-
Kato 2016 [43*]	US	68 / 122	32 / 41	AA: 14% in both gropus Rest: 86% others (primarily Caucasians)	No	No	-	Oral rinse	-
Kerdreux 2023 [57*] † FECSU cohort	Sweden	38 /63	20 / 33	-	-	-	Colonoscopy within 1 week, dementia and low performance status, including mental and physical disabilities	Feces	Before colonoscopy
U-CAN cohort ‡	Sweden	238 / 94	143 / 53	-	-	-	-	Feces	At the time of diagnosis and before starting treatment
Liu 2022 [58*] (Cohort AUS)	Austria	46 / 63	28 / 37	-	No	Yes (last 3 months)	-	Feces	Before colonoscopy
(Cohort CHN)	China	74 / 54	48 / 33	-	Yes	Yes (last 3 months)	Patients with hereditary CRC syndromes or previous history of CRC Healthy controls: without physician-diagnosed dementia, stroke, cancer, cardiovascular disease, psychiatric disorders or other serious illnesses	Feces	Before (Cases: 30; Controls:21) After (Cases:24; Controls:52)
(Cohort GER)	Germany	60 / 65	36 / 37	-	No	No	-	Feces	Cases: Before colonoscopy.Controls: withouth colonoscopy
Okumura 2021 [59*] (Cohort 1)	Japan	380 / 384	-	-	Yes	Yes	Patients: those whor received chemotherapy or radiation therapy before faecal sample collection, or faecal samples where collected after endoscopic tumour resection or CRC with a non adenocarcinoma tumour Both patients and controls: Those who had undergone gastrointestinal reconstructive surgery, severe hepatic dysfunction, with a faecal sample within 3 days of colonoscopy, or those without complete personal information	Feces	Before colonoscopy
(Cohort 2)	Japan	289 / 129	-	-	Yes	Yes		Feces	Before colonoscopy
Rezasoltani 2018 [60*]	Iran	20 / 31	12 / 16	-	Yes	Yes (last 6 months)	Using prebiotics and probiotics within the past 6 months1, vegetarian diet, an invasive medical intervention within 3 months (including endosonography, endoscopy, endoscopic retrogradecholangio-pancreatography and sphincterotomy within the past 3 months, history of any cancer of the intestine Controls: the above criteria and negative family history of gastrointestinal diseases	Feces	Before colonoscopy
Tarallo 2019 [44*]	Italy	29 / 24	-		Yes	No	-	Feces	Before colonoscopy
Vogtmann 2016 [61*]	US	52 / 52	37 / 37	Non-Hispanic white: 39 (75.0) / 47 (90.4) Non-Hisp. black & others: 13 25.0) / 5 (9.6)	No	No	-	Feces	Before colonoscopy
Wang 2021a [37*]	China	30 / 30	17 / 15	-	No	Yes (last month)	Prior history of nonsteroidal anti-inflammatory drug medication in 1-month, other types of malignant tumors	Feces, biopsy and saliva	Before and after colonoscopy
Wang 2021b [38*]	China	23 / 22	-	-	Yes	No ^#^	Known synchronous cancer diagnosis or other cancer diagnosis within 5 years of the operation	Feces	Prior to bowel preparation or 1 week after colonoscopy
Yang 2019 [63*]	US	231 / 461	93 / 185	EA: 53 (23.0)/ 106 (23.0); AA: 172 74.5)/ 343 (74.4); Others: 6 (2.6)/ 12(2.6)	No	Yes (last 7 days)	-	Mouth rinse	Before colonoscopy
Yang 2020 [62*] (Main cohort)	China	52 / 55	35 / 26	-	Yes	Yes	Patients: < 18 or > 76 years old; radiotherapy Controls: Metabolic disease (BMI > 32, diabetes or malnutrition), long term probiotics uptake Both: continuous treatment with systemic corticosteroids 1 month prior to sampling and serious mental disorder	Cases: feces, blood & urine Controls: feces	Before colonoscopy
(Validation cohort)	China	46 / 40	33 / 23	-	Yes	Yes		Cases: feces, blood & urine Controls: feces	Before colonoscopy
Zeller 2014 [64*]	France	53 / 61	29 / 28	-	Yes	No	Without previous colon or rectal surgery, CRC or need for emergency colonoscopy	Feces	Before colonoscopy

*CRC* Colorectal cancer, *FECSU cohort* Fecal and Endoscopic Colorectal Study in Urmeå, *U-CAN* Uppsala-Umeå Comprehensive Cancer Consortium, *AUS* Australia, *CHN* China, *GER* Germany, *EA* European-Americans, *AA* African-Americans

^#^No antibiotics were given only preoperatively

^##^*GCF* Gingival crevicular fluid

^†^14 patients were included in both cohorts (FECSU and U-CAN)

^‡^Controls in the U-CAN project were selected from the FECSU cohort with the same inclusion/exclusion criteria and density matched by age and gender

Among the exclusion criteria used to select participants, 60.9% (*n* = 14) of the studies excluded individuals who had taken antibiotics, particularly within the month preceding sample collection. Additionally, 60.9% (*n* = 14) of the included articles excluded participants with a history of gastrointestinal disease (e.g., acute or chronic diarrhea, irritable or inflammatory bowel disease, or hepatitis, among others). Table 1 reflects the variability of all other different exclusion criteria applied in 65.2% (*n* = 15) of the studies. Most studies (*n* = 16, 69.6%) analyzed PG only in fecal samples in the case–control analyses, three studies (13.0%) obtained only oral samples (saliva or rinse) and three analyses units (13.0%) collected multiple samples (feces, saliva and biopsy). Finally, another study (5.0%) collected fresh stool, blood and urine from patients and only fresh stool from healthy controls In 56.5% (*n* = 13) of studies, fecal samples were collected prior to colonoscopy, while only one study (5.3%) collected the sample after the procedure [40*]. Four studies (17.4%) collected samples both before and after colonoscopy. One study (4.3%) was at the time of diagnosis, and in three other studies (13.0%), the time of sample collection was not specified.

Table 2 describes the principal participants characteristics associated to factors known to be related to the microbiome composition and CRC development. Many study units provided data on mean age (13, 56.5%) and BMI (10, 43.5%). However, some did not report this information (10, 43.5%, and 13, 56.5%, respectively). or presented data in formats that precluded calculation of average values [56*], intervals [43*, 63*] or bar charts [59*]. The mean (standard deviation, SD) BMI was 25.4 (4.292) and 23.9 (1.606) among cases and controls, respectively. Only three analysis units (13.0%) reported periodontitis antecedents of participants [38*, 56*, 63*], five (21.7%) alcohol consumption [38*, 43*, 56*, 61*, 63*], six (26.1%) smoking status [38*, 43*, 56*, 60*, 61*, 63*], two (8.7%) physical activity [56*, 60*] and education [61*], one (4.3%) non-steroidal anti-inflammatory drug use [43*], diabetes mellitus [60*], oral hygiene [38*] and caffeine consumption and sleep disorders [56*]. Regarding CRC disease characteristics in cases, 13 analysis units reported both cancer stage and location of the cancer following the TNM or AJCC classification (Weiser, 2018).

**Table 2:** Description of included studies

								**CRC description**	
**Author, year**	**Age (mean)** **Cases/ Controls**	**BMI (mean)** **Cases/ Controls**	**Periodontal disease (%)** **Cases/** **Controls**	**Alcohol** **Consumption (%)** **Cases/** **Controls**	**Smoking (%)** **Cases/** **Controls**	**Physical activity (%)** **Cases/** **Controls**	**Other characteristics**	**Stage (%)**	**Location (%)**
Adel El-Sokkary 2022 [39*]	-	-	-	-	-	-	-	-	-
Conde-Pérez 2024 [56*]	-	-	58.1 / 50.0	32.3 / 30.1	15.0 / 20.0	43.0 / 23.3	Caffeine consumption: 19.3/29.4 Sleep disorders: 25.8/24.4	T1: 17.2; T2: 12.9; T3: 62.4; T4: 5.4; N0: 64.5; N1: 17.2; N2: 7.5; N3:2.1; N4: 2.1; N5: 2.1; M0: 92.5; M1: 2.1; M2: 2.1; M3: 1.1	Cecum: 6.4; Ascending colon: 31.2; Hepatic-transvers-splenic: 6.4; Descending colon: 23.6; Sigmoid colon: 19.3; Rectum: 10.7; Undetermined: 2.1
Fukugaiti 2015 [41*]	65.4 / 54.8	-	-	-	-	-	-	-	-
Gao 2020 [40*]	65.8 / 65.0	23.2 / 23.4	-	-	-	-	-	0 + I: 16.1; II: 32.9; III: 36.1; IV: 7.1; Undefined: 7.7	Ascending colon: 19.3; Transverse colon: 4.5; Descending colon: 6.4; Sigmoid colon: 21.3; Rectum: 45.2; Undefined: 2.2
Gao 2021 [36*]	61.9 / 60.2	22.9 / 23.3	-	-	-	-	-	I: 12.7; II: 42.3; III: 38.0; IV: 7.0	Ascending colon: 22.5; Transversal colon: 7.0; Descending colon: 2.8; Sigmoid colon: 11.3; Rectum: 56.3
Guven 2019 [42*]	59.0 / 56.0	-	-	-	-	-	-	I: 5.6; II: 22.5; III: 33.8; IV: 38.0	Right: 22.5; Left: 77.5
Kato 2016 [43*]	-	-	-	< 1 week: 55.9 / 64.9 1–6 w: 27.9 / 23.7 > 7 w: 16.2 / 11.4	Never: 38.2 / 40.2 Ex: 38.2 / 30.3 Current: 23.5 / 29.5	-	Non-steroidal anti-inflammatory drug use: 20.6 / 32.0	-	
Kerdreux 2023 [57*]^a, b^ FECSU cohort	-	-	-	-	-	-		I: 5.4; II: 54.1; III: 21.6; IV: 7	Right colon: 28.9; Left colon: 44.7; Rectum: 26.3
U-CAN cohort^c^	-	-	-	-	-	-		I: 20.4; II: 34.2; III: 28.9; IV: 16.4	Right colon: 20.2; Left colon: 17.2; Rectum: 62.6
Liu 2022 [58*] (Cohort AUS)	67.1 / 67.1	26.7 / 27.6	-	-	-	-	-	0: 15.6; I/II: 57.8; III/IV: 26.7	Left: 23.9; Right: 17.4; Rectum: 58.7
(Cohort CHN)	66.0 / 61.8	24.0 / 23.5	-	-	-	-	-	I/II: 54.1; III/IV: 45.9	Left: 35.1; Right: 14.9; Rectum: 37.8; Others: 12.2
(Cohort GER)	63.5 / 56.0	26.2 / 24.9	-	-	-	-	-	0: 5.0; I/II: 58.3; III/IV: 36.7	Left: 26.7; Right: 25.0; Rectum: 43.3; Others: 5.0
Okumura 2021 [59*] (Cohort 1)	-	-	-	-	-	-		-	-
(Cohort 2)	-	-	-	-	-	-	-	-	-
Rezasoltani 2018 [60*]	69.9 / 58.8	-	-		6.3 / 22.6	No: 81.2 / 67.7 Low: 12.5 / 22.6 High: 6.3 / 9.7	Diabetes Mellitus: 25.0 / 6.5	-	-
Tarallo 2019 [44*]	-	-	-	-	-	-	-	-	-
Vogtmann 2016 [61*]	61.8 / 61.2	24.9 / 25.3	-	Drinks/wk: 7.4 / 6.1	Never: 46.2 / 42.3; Former: 34.6 / 53.8; Current: 13.5 / 3.8; Missing: 5.8 / 0	-	Education: Less than high school: 15.4/3.8; High school graduate: 21.2/19.2; 1–5 years of college/graduate: 42.3/46.1; 6 + years of college/graduate: 15.4/30.8	Preinvasive: 23.1; Invasive, no known metastases: 40.4; Known metastases: 34.6; Missing data: 1.9	Right colon: 28.8; Left colon: 34.6; Rectal: 26.9; Missing data: 9.6
Wang 2021a [37*]	64.0 / 52.2	23.6 / 23.2	40.0 / 46.7	20.0 / 33.3	13.3 / 13.3		Higher oral hygiene index (OHI) (median): 2/1	-	-
Wang 2021b [38*]	-	-	-	-	-	-	-	-	-
Yang 2019 [63*]	-	-	Gingivitis: 13.4 7 18.89	None: 60.3 7 / 49.8; Light: 22.9 / 31.0; Moderate: 7.9 / 11.6; Heavy: 8.8 / 7.6	Current: 27.7 / 27.8 Former: 32.0 / 32.1; Never: 40.3 7 40.1;	-	Education: < High School: 25.6/27.3; High/Vocational School: 37.0/35.9; Some college: 19.6/17.5; College: 17.8/19.3	-	-
Yang 2020 [62*] (Main cohort)	53.0 / 42.0	23.4 / 23.2	-	-	-	-	-	I: 25.0; II: 34.6; III: 30.8; IV: 9.6	Colon: 44.2; Rectum: 55.8
(Validation cohort)	59.0 / 42.5	22.5 / 22.8	-	-	-	-	-	I: 21.7; II: 39.1; III: 32.6; IV: 6.5	Colon: 37.0; Rectum: 63.0
Zeller 2014 [64*]	64 / 63.0	37.0 / 22.0	-	-	-	-	-	TNM: T1: 17.0; T2: 15.1; T3: 45.3; T4: 22.6; N0: 48.1; N1: 51.9; N2: 0.0; N3: 0.0; N4: 0.0; N5: 0.0; M0: 60.4; M1: 39.6; M2: 0.0; M3: 0.0 AJC Stage: I: 26.4; II: 13.2; III: 18.9; IV: 41.5	Right colon: 32.1; Left colon: 43.4; Sigma: 7.5; Rectum: 17.0

*FECSU cohort* Fecal and Endoscopic Colorectal Study in Urmeå, *U-CAN* Uppsala-Umeå Comprehensive Cancer Consortium, *AUS* Australia, *CHN* China, *GER* Germany

^a^14 patients were included in both cohorts (FECSU and U-CAN)

^b^Cancer description from Lövenmark et al. (2020)

^c^Controls in the U-CAN project were selected from the FECSU cohort with the same inclusion/exclusion criteria and density matched by age and gender

### Main results

Most studies (*n* = 17; 73.9%) reported their results in terms of relative abundance (see Table 3). Different methods were used to detect PG in samples, the majority of studies (*n *= 14, 60.1%) employed sequencing techniques. Of these, 64.3% (*n* = 9) used 16 s rRNA sequencing, and the remainder conducted metagenomic sequencing (see Table 3). Nine analysis units (39.1%) used PCR based techniques (PCR/rtPCR/qPCR).

**Table 3:** Main results of included studies

Author, year	Detection method	Quatification measures reported	Results in cases	Results in controls	p-value original	Sign	P-value analysis	Detección P.gingivalis
Adel El-Sokkary, 2022 [39*]	Real-time PCR	Number of positive measures (%)	10 (37%)	0 (0%)	-	+	.1231^b^	Increased
Conde-Pérez, 2024 [56*]	16S rRNA gene sequencing	Relative abundance	(*)	(*)	n.s	-	-	Increased^a^
Fukugaiti, 2015 [41*]	qRT-PCR	Number of positive measures (%)	7 (100%)	10 (100%)	.5	+	.2500	Normal
Gao, 2020 [40*]	16 s rRNA sequencing	Relative abundance	(*)	(*)	-	-	-	Increased
Gao, 2021 [36*]	DNA metagenomic sequencing	Relative abundance	(*)	(*)	-	-	-	Increased
Guven, 2019 [42*]	Real-time PCR	Number of positive measures (%)	54 (76.1%)	58 (75.3%)	.917	-	.5415	Normal
Kato, 2016 [43*]	16 s rRNA sequencing	Relative abundance	(*)	(*)	-	-	-	Normal
Kerdreux, 2023 [57*] FECSU cohort	Real-time PCR	Relative abundance	1 (38%)	0 (0%)	n.s	-	-	No differences
U-CAN cohort	Real-time PCR	Relative abundance	13 (5.3%)	0 (0%)	.028	+	.0140	Increased
Liu, 2022 [58*] (Cohort AUS)	Shotgun metagenomic sequencing	Relative abundance	(*)	(*)	.00008	+	.00004	Increased
(Cohort CHN)	Shotgun metagenomic sequencing	Relative abundance	(*)	(*)	.5116	+	.2558	Increased
(Cohort GER)	Shotgun metagenomic sequencing	Relative abundance	(*)	(*)	.153	+	.0765	Increased
Okumura, 2021 [59*] (Cohort 1)	16 s rRNA sequencing	Relative abundance	(*)	(*)	.000259	+	.00013	Increased
(Cohort 2)	16 s rRNA sequencing	Relative abundance	(*)	(*)	.425 (^a^) .0297 (^b^)	+ +	.2125 .01485	Increased
Rezasoltani, 2018 [60*]	Real-time PCR	Abundance	Mean (SD) = 24.58 (7.36)	Mean (SD) = 27.96 (2.75)	-		-	Increased
Tarallo, 2019 [44*]	16 s rRNA sequencing	Relative abundance	Median = .03; Average = .05	Median = .05; Average = .09	.0798	-	.9601	Diminished
Vogtmann, 2016 [61*]	16 s rRNA sequencing	Relative abundance	Mean relative abundance = 6.89E-06;	Mean relative abundance = 1.81E-06;	.4980	+	.2490	Increased
Wang, 2021(a) [37*]	16 s rRNA sequencing	Abundance	(*)	(*)	-		-	Increased
Wang, 2021(b) [38*]	Q real-time PCR	Relative abundance	(*)	(*)	< .01		-	Increased
Yang, 2019 [63*]	16 s rRNA sequencing	Relative abundance	150 (64.9%)	296 (64.2%)	.8	-	.6000	Increased
Yang, 2020 [62*] (Cohort)	qPCR	Relative abundance	.002347	0.000641	.0216			Increased
(Validation Cohort)	qPCR	Relative abundance	.004457	0.002046	.0216	+	.01078	Increased
Zeller, 2014 [64*]	Metagenome sequencing	Abundance	2 (3.8%)	2 (3.3%)	NA	+	.4427^b^	Increased

*FECSU cohort* Fecal and Endoscopic Colorectal Study in Urmeå; *U-CAN* Uppsala-Umeå Comprehensive Cancer Consortium, *AUS* Australia, *CHN* China, *GER* Germany

(*) Results are presented in a graphic; (a) early CRC cases compared to healthy controls; (b) advanced CRC cases compared to healthy controls

^a^P.G. relative abundance was measured, however, evaluation of the detection was presented as *Porphyromonas* in general

^b^Calculated using frequencies reported;

n.s.: not statistical significance

Of the 23 analysis units, 18 of them (78.3%) found an increased detection of PG in cases of CRC compared to healthy controls, 4 (17.4%) found no difference or a normal concentration and the PG concentration was diminished in only 1 unit (4.3%) [44*]. However, only 13 units (56.5%) provided p-values testing the association between PG and CRC, and only 6 of them were significant providing an increased concentration (26.1%) were significant.

Using Fisher’s method to combine these yielded a result of ${\chi }_{30}^{2}$ = 88.84165; *p* = 0.0000001, indicating evidence of an effect in at least one study. The sign test based on the same dataset yielded a value of Z = 2.065591; *p* = 0.038, suggesting some evidence of a higher concentration of PG in the gut microbiota of CRC cases compared to healthy controls. Sensitivity analyses removing one result to address dependencies (Okumura 2021, early CRC cases, shared control group) yielded similar results (${\chi }_{28}^{2}$ = 85.74402; *p* = 0.0000001 for the combination of p-values, and Z = 1.870829; *p *= 0.061) for the sign test based on vote counting.

### Risk of bias analyses

**Fig. 2:**
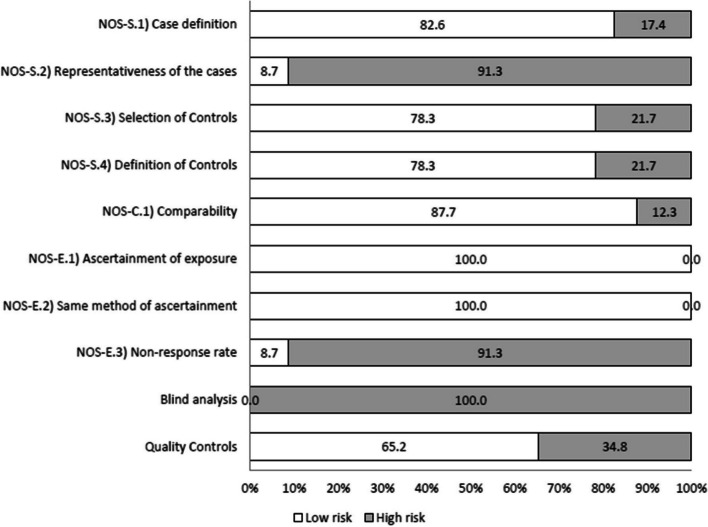
Risk of bias assessment according to the NOS (Newcastle-Otawa Scale)

Table 4 presents the results of the risk of bias assessment for the included studies, conducted using the NOS scale along with two additional criteria. The average NOS score was 6.4 (SD: 1.4, range: 2 to 8) of a total of 9. According the Agency for Healthcare Research and Quality (AHRQ), 15 analyses units (65.2%) were classified as good quality, 3 (13.0%) as acceptable and 5 (21.7%) as poor quality. Nearly all analyses units (*n* = 20; 87.0%) were adjusted for more than one variable, generally age and BMI. However, only two studies controlled for smoking [38, 63*], and only one accounted for alcohol consumption [38] and dietary patterns, such as vegetarianism [60*]. The factors with the highest risk of bias (greater than 50%) were the representativeness of the cases (NOS-S.2) and the absence of information regarding response rates and participation attrition (NOS-E.3), as illustrated in Fig. 2. Furthermore, none of the studies reported conducting analyses blinded to case–control status, and the majority (*n* = 15; 65.2%) used internal methods to assess the quality of laboratory analyses. According to the GRADE system [51], current certainty of evidence for the association between PG with CRC is very low. This assessment was based on the observational nature of the study designs (initially rated as Low) and was further downgraded according to GRADE criteria (see Supplementary Table 3).

**Table 4:** Individual risk of bias assessment according to NOS (Newcastle–Ottawa Scale) for case–control studies

	**SELECTION**				**COMPARABILITY**	**EXPOSITION**				
First autor, year of publication	Case definition	Representativeness of cases	Selection of controls	Definition of controls	Comparability	Ascertainement of exposure	Ascertainment method for cases and controls	Non-response rate	Blind analysis	Quality control
Adel El-Sokkary, 2022 [39*]	-	-	-	-	-	*	*	-	No	No
Conde-Pérez, 2024 [56*]	*	-	-	*	-	*	*	-	No	Yes
Fukugaiti, 2015 [41*]	*	-	*	*	**	*	*	-	No	No
Gao, 2020 [40*]	*	-	*	*	**	*	*	-	No	Yes
Gao, 2021 [36*]	*	-	*	*	**	*	*	-	No	Yes
Guven, 2019 [42*]	*	-	*	*	*	*	*	-	No	No
Kato, 2016 [43*]	-	*	*	-	**	*	*	*	No	No
Kerdreux, 2023^a^ [57*] FECSU cohort	*	-	*	*	**	*	*	-	No	No
U-CAN cohort^b^	*	-	-	*	**	*	*	-	No	No
Liu, 2022 [58*]										
(Cohort AUS)	*	-	*	*	**	*	*	-	No	Yes
(Cohort CHN)	*	-	*	*	**	*	*	-	No	Yes
(Cohort GER)	*	-	-	-	**	*	*	-	No	Yes
Okumura, 2021 [59*]										
(Cohort 1)	*	-	*	*	**	*	*	-	No	Yes
(Cohort 2)	*	-	*	*	**	*	*	-	No	Yes
Rezasoltani, 2018 [60*]	*	-	*	*	**	*	*	-	No	No
Tarallo, 2019 [44*]	*	-	*	*	**	*	*	-	No	Yes
Vogtmann, 2016 [61*]	-	-	-	-	**	*	*	-	No	No
Wang, 2021a [37*]	*	-	*	*	**	*	*	-	No	Yes
Wang, 2021b [38*]	*	-	*	*	-	*	*	-	No	Yes
Yang, 2019 [63*]	-	*	*	-	**	*	*	*	No	Yes
Yang, 2020 [62*]										
(Main Cohort)	*	-	*	*	**	*	*	-	No	Yes
(Validation Cohort)	*	-	*	*	**	*	*	-	No	Yes
Zeller, 2014 [64*]	*	-	*	*	**	*	*	-	No	Yes

*FECSU cohort* Fecal and Endoscopic Colorectal Study in Urmeå, *U-CAN* Uppsala-Umeå Comprehensive Cancer Consortium, *AUS* Australia, *CHN* China, *GER* Germany

^**a**^14 patients were included in both cohorts (FECSU and U-CAN)

^b^Controls in the U-CAN project were selected from the FECSU cohort with the same inclusion/exclusion criteria and density matched by age and gender

### Potential sources of heterogeneity

Since a meta-analysis could not be performed, a formal heterogeneity analysis could not be implemented either. However, to detect potential sources of heterogeneity, the number and percentage of studies with the main characteristics by increased PG concentration detected in CRC patients compared to controls is presented in Table 5. Only reporting participants’ BMI was significatively associated to an increase detection of PG concentration in CRC patients.

**Table 5:** Description of items by subgroups

	**Total**	**Not increased**	**Increased**
**Number of Unit of Analysis (%) #**	23	5 (21.7)	18 (78.3)
**Exclusion criteria in participants**			
Antecedents of digestive tract diseases			
No	9 (39.1)	2 (40.0)	7 (38.9)
Yes	14 (60.9)	3 (60.0)	11 (61.1)
Prior antibiotic use			
No	9 (39.1)	3 (60.0)	6 (33.3)
Yes	14 (60.9)	2 (40.0)	12 (66.7)
**Biological samples**			
Feces	16 (69.6)	3 (60.0)	13 (72.2)
Oral	3 (13.0)	2 (40.0)	1 (5.6)
Mixed	4 (17.4)	-	4 (22.2)
**Sample collection time related to colonoscopy**			
Before	13 (65.0)	3 (100.0)	10 (58.8)
After or mixed	7 (35.0)	-	7 (41.2)
**Quantification measures reported**			
Nº positive measures	3 (13.0)	2 (40.0)	1 (5.6)
Relative abundance	20 (87.0)	3 (60.0)	17 (94.4)
**High risk of bias (RoB) > 25%**			
NOS-S.3) Representativeness cases			
No	19 (82.6)	3 (60.0)	16 (88.9)
Yes	4 (17.4)	2 (40.0)	2 (11.1)
NOS-E3) Reporting non-response rate			
No	21 (91.3)	4 (80.0)	17 (94.4)
Yes	2 (8.7)	1 (20.0)	1 (5.6)
Quality assessment in analyses			
No	8 (34.8)	4 (80.0)	4 (22.2)
Yes	15 (65.2)	1 (21.7)	18 (78.3)

Values in brackets are percentages by column (within each column)

## Discussion

To the best of our knowledge, this study represents the most comprehensive exploration to date of the specific relationship between *Porphyromonas gingivalis* (PG) and CRC. This systematic review synthesizes findings from 18 case–control studies comprising 23 units of analysis that assessed the presence of PG in CRC patients compared to healthy controls. The results indicate that PG concentrations are higher in CRC patients compared to controls. Additionally, several of the included studies also reported associations between PG and other adenomas [36*, 37*, 40*, 59*, 60*], as well as with the risk of developing adenomatous polyp and polyp size [60, 24], digestive cancer [65], in particular gastrointestinal cancer [12]. In these studies, PG was identified as one of the most prevalent pathogens in the gut microbiota of CRC patients. Our results also align with evidence linking other oral pathogens to CRC. For instance, several meta-analyses have suggested that *Fusobacterium nucleatum* (FN), one of the most prevalent oral pathogens implicated in periodontitis, is associated with CRC compared to healthy controls [29–32], particularly as a risk factor for metastatic CRC [28]. Another oral pathogen, *Parvimonas micra*, has also been associated with CRC [66, 67]. Periodontal bacterial infections appear to increase cancer incidence [25], and this association is particularly evident in CRC [68].

This systematic review also underscores the need for careful consideration of potential confounders and sources of variability. Case–control studies included in this review exhibited relevant heterogeneity in measurement and reporting methods (e.g., sample type, timing of collection- pre- vs. post-colonoscopy, and sequencing technologies) which precluded the possibility of conducting a meta-analysis. Among the analyzed variables, only reporting BMI showed a significant association with higher PG concentration in CRC patients with obesity (BMI ≥ 25 kg/m^2^). Obesity is recognized as a strong risk factor for CRC [69, 70]; however, less than 45% of the analysis units measured BMI data. However, the small number of analysis units reporting other variables warrants a cautious interpretation of these findings, regardless of their statistical significance. Several studies suggest that patients with inflammatory bowel disease [71, 72], chronic liver diseases [73], and systemic chronic inflammation [74] may have an increased risk of CRC. Furthermore, excluding participants who used antibiotics prior to sample collection seems reasonable, as antibiotics not only alter gut microbiota composition [75], but have also been associated with an increased risk of CRC with cumulative use [76]. Probiotics may play a protective role in preventing CRC [77]. However, only 14 analysis units (60.9%) considered digestive diseases and prior antibiotic use as exclusion criteria, while just 5 (21.7%) included probiotics or prebiotics use as a criterion for participant selection. This omission represents a potential source of selection bias.

Similarly, ethnicity, a factor influencing microbiome composition [78] and CRC association, was reported in only three analysis units (13.0%). For instance, FN has shown a stronger association with CRC in Asian population compared to American or European populations [30]. Other important CRC risk factors, such as smoking [79, 80] and alcohol consumption [81, 82] were also infrequently reported. It is notable that only three studies (13.0%) assessed a history of periodontal diseases, despite the focus on one of the most prevalent periodontal pathogens. Moreover, just two (8.7%) examined physical activity, a healthy lifestyle known to influence gut microbiota [83, 84] and considered as a protective factor against CRC [85]. Critical information, such as the timing of sample collection, were not reported in some studies [39*, 42*, 43*] and in one case [40, 86–88]. Additionally, studies employed various sequencing technologies have been used. Advances in genomic research over the past decades have introduced newer sequencing methods [89, 90]. Special attention should be paid to the sequencing technologies used as differences in quality, diversity and abundance of microbial data between them have been reported [91, 92].

Risk of bias assessment indicated that most studies were of good or acceptable quality, according to the Agency for Healthcare Research and Quality (AHRQ) criteria [17, 30, 50]. However, when evaluated using GRADE criteria [51], the certainty of evidence was deemed very low. This was primarily due to the observational nature of the study designs and their inherent limitations to support causal relations. Several key issues require greater attention in future research. Specifically, the representativeness of the cases was often inadequately addressed, as many studies failed to specify whether all eligible cases with the outcome of interest were included, or if they were selected from a defined healthcare center. Additionally, information on the non-response rate for both cases and controls was frequently lacking.

Several strengths of our study should be highlighted. First, the study protocol was pre-registered in PROSPERO, ensuring transparency and methodological rigor. Second, quality assessment and risk of bias [93], as well as the quality of evidence, were assessed using the NOS [49] and GRADE criteria [51]. Third, two researchers independently conducted the study selection and data extraction processes, with disagreements resolved by consensus or participation of a third reviewer. Nevertheless, some limitations at both the study and review levels should be carefully considered. At the study level, two primary issues were identified. First, the data extraction process was complex and challenging due to incomplete reporting. However, the use of a pre-defined and registered protocol, along with independent coding by two reviewers, mitigated some of these challenges. Adoption of reporting guidelines, such as the Strengthening the Reporting of Observational Studies in Epidemiology (STROBE) statement [94], specifically aimed at improving the quality of reporting in observational studies, could greatly facilitate data extraction in future research. Second, the presence of PG in CRC patients may have been underestimated, as we only included studies that specifically provided species-specific data on PG data. Several excluded studies reported microbiota findings only at the genus level (*Porphyromonas*), without species-level details. Previous systematic reviews [25, 65, 95–97] and other excluded studies [98–101] have also identified significantly elevated levels of the *Porphyromonas* genus in CRC cases. Future research should focus on identifying specific altered species associated with CRC, also including other members of the *Porphyromonas* genus, such as *Porphyromonas asaccharolytica*, which may also contribute to CRC development [102].

At the review level, the main limitations were the scarce number of included studies and the heterogeneity in their analytical methods and result presentation. This variability precluded the use of meta-analysis, leading us to adopt Fisher’s method for combining p-values instead. While this approach offers a more robust alternative to narrative synthesis, it has notable limitations. It does not provide information on the magnitude of the effect size, cannot distinguish between evidence from large studies with small effects and small studies with large effects, and, finally, makes the interpretation of test results more challenging [53]. The development of standardized and reproducible protocols will greatly facilitate forthcoming meta-analysis. These protocols should include clear inclusion and exclusion criteria (e.g., history of gastrointestinal diseases, prior use of antibiotics, prebiotics or probiotics, or other treatment affecting gut microbiota, among others), standardized sample handling procedures (e.g., collection methods, storage conditions, and duration), consistent analytical platforms, and effect size measures. Additionally, protocols should identify a core set of confounding factors to control for, such as BMI, dietary habits, alcohol consumption or smoking [65, 103]. Implementing such protocols will reduce risk of bias and heterogeneity across studies, thereby enhancing the reliability and comparability of findings. Consequently, these efforts will improve the feasibility and robustness of future meta-analyses.

PG is a Gram-negative, anaerobic oral bacterium, and a major pathogen in severe adult periodontitis [33, 34]. It has been implicated in gut dysbiosis and in the CRC development [26, 104, 105]. This raises two critical questions: how does this oral pathogen disseminate to distant organs, and what are the underlying pathogenic mechanisms driving its role in CRC? One hypothesis is that bacterial toxins or metabolites produced by PG may promote carcinogenesis at distant sites [105]. However, the detection of PG in the gut microbiome indicates direct colonization of the colon. While exogenous sources (e.g., environmental exposure) may contribute, evidence strongly supports endogenous colonization originating from the oral cavity. This likely occurs through the continuous swallowing of oral bacteria or via bacteremia with PG surviving the acidic gastric environment of the stomach to reach the colon [26]. The exact mechanism by which PG contributes to CRC remains unclear, though several pathogenic pathways have been proposed. For instance, PG may modulate the immune response, fostering dysbiosis and promoting cancer development [106]. Periodontitis triggers a robust immune response, including elevated pro-inflammatory cytokines, increased serum C-reactive protein (CRP), and reduced anti-inflammatory markers such as interleukin-10 [24]. Other proposed mechanisms include the induction of cellular senescence through butyrate secretion [59, 107]; and recruitment of myeloid cells alongside activation of the hematopoietic NLRP3 (Nod-like receptor pyrin domain-containing 3) inflammasome [37], among others [106]. Together, these findings suggest that PG acts as a potential pro-oncogenic organism with significant implications in CRC pathogenesis. However, these molecular mechanisms may be PG-specific, underscoring the need for further species-specific research [105]. Interestingly, a recent meta-analysis suggests that the pathogenic effects of PG may extend beyond CRC, contributing to increased incidence and poorer prognosis in other malignancies [25].

Our systematic review aimed to elucidate the association between PG and CRC by comparing cases with healthy controls. Consequently, studies focused on CRC prognosis and validation of screening or diagnostic tests were excluded. Nonetheless, follow-up studies of CRC cohorts have consistently reported elevated PG levels in CRC patients [108], with associations observed in metastasis [100, 109], early-onset [110], poorer prognosis [37], and higher mortality of CRC [21]. Furthermore, several studies have explored PG’s potential as a noninvasive biomarker for detecting precancerous colorectal polyps [111], and early CRC diagnosis [112], yielding promising results [36, 97, 101, 40*, 60*, 62*, ]. Collectively, these findings suggest that the detection of *Porphyromonas* species, particularly PG, alongside other pathogens, could enhance current screening methods, such as the fecal occult blood test (FOBT) [113], for detecting precancerous and cancerous colorectal lesions. This approach may also aid in early detection and prevention of other gastrointestinal cancers [12].

Additionally, it is crucial to examine interactions among gut bacteria, fungi, and viruses in CRC [36, 114, 115, 58*, ], as well as the influence of host factors such as diet, lifestyle, and medication use [112]. These complex interactions emphasize the need for well-designed studies with larger and more diverse populations to elucidate PG’s causal, prognostic, and diagnostic roles in CRC. A deeper understanding of the pathophysiological mechanisms and interactions between *Porphyromonas gingivalis* (PG), the gut microbiota, and host systems is essential for advancing CRC prevention and improving patient prognosis [116]. Primary preventive strategies should include maintaining good oral hygiene, treating periodontal diseases, adopting healthy diets, and the use of prebiotics, probiotics, and postbiotics [117]. In the context of rising antibiotic resistance, novel approaches such as bacteriophage therapies [118], antimicrobial photodynamic therapy [119], PG vaccines [120], and as well as fecal microbiota transplantation (FMT) [117] offer promising avenues for CRC prevention and treatment.

## Conclusions

In summary, despite the limited current evidence, PG, an oral bacterium closely related to periodontitis, appears to be associated with CRC. This finding aligns with existing literature and highlights the importance of improving oral health and microbiota control as key strategies for the primary prevention of CRC. Furthermore, PG represents a promising therapeutic target for improving CRC prognosis. However, larger prospective cohort studies, with specific protocols aimed at reducing heterogeneity across studies, are necessary to better understand the complex relationships between dysbiosis and CRC. The development of standardized and reproducible protocols will be instrumental in advancing the study of the human gut microbiome, which could lead to significant breakthroughs in personalized medicine and disease prevention. By harmonizing analyses and effect size measurements, these efforts will reduce heterogeneity and make results more comparable, facilitating future meta-analyses. It is critical to clarify the specific role of the individual microorganisms involved in CRC pathogenesis, as well as their potential interactions. Our study suggests that PG can be considered as a promising modifiable tumorigenic factor and highlights its future role as a potential biomarker for diagnoses, progression, recurrence, and therapeutic target of CRC.

## Supplementary Information

Supplementary Material 1: Table 1. Search strategy used by databases. Table 2. Table of excluded studies. Table 3. Grading of Recommendations Assessment, Development and Evaluation (GRADE) assessment.

## Data Availability

All relevant data are available from within the manuscript as well as the supporting information.

## Abbreviations

AHRQ: Agency for Healthcare Research and Quality
BMI: Body mass index
CRC: Colorectal cancer
GRADE: Grading of Recommendations Assessment, Development and Evaluation
FOBT: Feccal Occult Blood Test
FMT: Fecal Microbiota Transplantation
NOS: Newcastle-Ottawa Scale
PG: *Porphyromona Gingivalis*
PCR: Polymerase Chain Reaction
PRISMA: Preferred Reporting Items for Systematic Reviews and Meta-Analyses
PROSPERO: International Prospective Register of Systematic Reviews
STROBE: Strengthening the Reporting of Observational Studies in Epidemiology
SWIM: Synthesis without Meta-analysis
TQS: Total quality score
