# Supplementary material for: Is the oral pathogen, Porphyromona gingivalis, associated to colorectal cancer?: a systematic review
Source: BMC Cancer. 2025 Mar 4;25:395. doi: 10.1186/s12885-025-13770-4 (PMC11881450; doi:10.1186/s12885-025-13770-4)
Supplement: Supplementary file 1 — Supplementary Material 1: Table 1. Search strategy used by databases. Table 2. Table of excluded studies. Table 3. Grading of Recommendations Assessment, Development and Evaluation (GRADE) assessment. [file 12885_2025_13770_MOESM1_ESM.docx]

## Supplementary Table 1. Search strategy used in each of the databases.

| DATABASES | | 1^st^ search | Update |
| --- | --- | --- | --- |
| **MEDLINE** | | | |
| 1 | *colorectal cancer OR colon cancer OR rectal cancer OR cancer of the colorectum OR cancer of the colon OR cancer of the rectum OR colorectal neoplasm OR colon neoplasm OR rectal neoplasm OR colorectal tumour OR colon tumour OR rectal tumour OR colorectal carcinoma OR colon carcinoma OR rectal carcinoma* |  |  |
| 2 | *Porphyromonas OR Porphyromonas Gingivalis* |  |  |
| 3 | 1 and 2 | 79 | 43 |
| **EMBASE** | | | |
| 1 | *colorectal cancer OR colon cancer OR rectal cancer OR cancer of the colorectum OR cancer of the colon OR cancer of the rectum OR colorectal neoplasm OR colon neoplasm OR rectal neoplasm OR colorectal tumour OR colon tumour OR rectal tumour OR colorectal carcinoma OR colon carcinoma OR rectal carcinoma* |  |  |
| 2 | *Porphyromonas OR Porphyromonas Gingivalis* |  |  |
| 3 | 1 and 2 | 188 | 29 |
| **WEB OF SCIENCE** | | | |
| 1 | *((((((((((((((TS=(Colorectal cancer)) OR TS=(colon cancer)) OR TS=(rectal cancer)) OR TS=(cancer of the colorectum)) OR TS=(cancer of the colon)) OR TS=(cancer of the rectum)) OR TS=(colorectal neoplasm)) OR TS=(colon neoplasm)) OR TS=(colorectal tumour)) OR TS=(colon tumour)) OR TS=(rectal tumour)) OR TS=(colorectal carcinoma)) OR TS=(colon carcinoma)) OR TS=(rectal carcinoma))* |  |  |
| 2 | *(TS=(Porphyromonas) OR TS=(Porphyromonas Gingivalis))* |  |  |
| 3 | 1 and 2 | 205 | 101 |
| **SCOPUS** | | | |
| 1 | *Colorectal AND cancer OR colon AND cancer OR rectal AND cancer OR cancer AND of AND the colorectum OR cancer AND of AND the AND colon OR cancer AND of AND the AND rectum OR colorectal ANS neoplasm OR colon AND neoplasm OR rectal AND neoplasm OR colorectal AND tumour OR colon AND tumour OR rectal AND tumour OR colorectal AND carcinoma OR colon AND carcinoma OR rectal AND carcinoma* |  |  |
| 2 | *porphyromonas OR porphyromonas AND gingivalis* |  |  |
| 3 | 1 and 2 | 760 | 74 |
| **TOTAL** | | 1232 | 247 |

Supplementary table 2. List of excluded studies with the main reason for exclusion.

| **First author** | **Year** | **Journal** | **Doi** | **Reason for exclusion** |
| --- | --- | --- | --- | --- |
| 1. Abdi et al | 2022 | Jundishapur | 10.5812/jjm-121119 | No *P. gingivalis* analyses |
| 1. Ahn et al | 2012 | Carcinogen | 10.1093/carcin/bgs112 | No healthy controls |
| 1. Ahn et al | 2013 | Journal of the National Cancer Institute | 10.1093/jnci/djt300 | No *P. gingivalis* analyses |
| 1. Ai et al | 2019 | Frontiers in Microbiology | 10.3389/fmicb.2019.00826 | No original data |
| 1. Ai et al | 2019 | Genes | 10.3390/genes10020112 | No original data |
| 1. Ai et al | 2019 | PeerJ | 10.7717/peerj.7315 | No original data |
| 1. Alexander et al | 2017 | Gastroenterology | [10.1016/S0016-5085(17)33427-3](http://dx.doi.org/10.1016/S0016-5085(17)33427-3) | Poster/Abstract |
| 1. Allali et al | 2018 | Med. Microbiol. Immunol. | 10.1007/s00430-018-0542-5 | No *P. gingivalis* analyses |
| 1. Alomair et al | 2018 | Gastroenterology Research and Practice | 10.1155/2018/5284754 | No *P. gingivalis* analyses |
| 1. Amini et al | 2024 | J Gastrointest Cancer | 10.1007/s12029-023-00962-z | Different outcome |
| 1. An et al | 2023 | Front Oncol | 10.3389/fonc.2023.1310054 | No health controls |
| 1. Baxter et al | 2016 | Genome | 10.1186/s13073-016-0290-3 | No healthy controls |
| 1. Cai et al | 2023 | Fron Cell Infect Microbiol | 10.3389/fcimb.2023.1123544 | No healthy controls |
| 1. Chen et al | 2012 | Plus ONE | 10.1371/journal.pone.0039743 | No *P. gingivalis* analyses |
| 1. Chen et al | 2022 | Gut Pathogens | 10.1186/s13099-022-00527-8 | No original data |
| 1. Dai et al | 2018 | Microbiome | 10.1186/s40168-018-0451-2 | No *P. gingivalis* analyses |
| 1. Dai et al | 2018 | Gastroenterology | 10.1371/journal.pone.0039743 | Poster/Abstract |
| 1. Diaz-Basabe et al | 2024 | Gut Microes | 10.1080/19490976.2024.2388801 | No healthy controls |
| 1. Du et al | 2022 | Frontiers in Physiology | 10.3389/fphys.2022.854545 | No *P. gingivalis* analyses |
| 1. Einaggar et al | 2023 | Fron Immunol | 10.3389/fimmu.2023.1051431 | No healthy controls |
| 1. Eklöf et al | 2017 | International Journal of Cancer | 10.1002/ijc.31011 | No *P. gingivalis* analyses |
| 1. Feng et al | 2015 | Nature Communications | 10.1038/ncomms7528 | No *P. gingivalis* analyses |
| 1. Flemer et al | 2017 | Gut | 10.1136/gutjnl-2015-309595 | No *P. gingivalis* analyses |
| 1. Flemer et al | 2018 | Gut | 10.1136/gutjnl-2017-314814 | No *P. gingivalis* analyses |
| 1. Fu et al | 2024 | Discov Oncol | 10.1007/s12672-024-01393-3 | No healthy controls |
| 1. Galán Ros et al | 2015 | Clinical Chemistry and Laboratory Medicine | 10.1515/cclm-2015-5033 | Poster/Abstract |
| 1. Geng et al | 2014 | Gut Pathogens | 10.1186/1757-4749-6-26 | No *P. gingivalis* analyses |
| 1. Guven et al | 2018 | J. Clin. Oncol. | 10.1200/JCO.2018.36.15-suppl.e15617 | Poster/Abstract |
| 1. Gu et al | 2024 | Front. Cell. Infect. Microbiol. | 10.3389/fcimb.2024.1452392 | No original data |
| 1. Kasai et al | 2016 | Oncology Reports | 10.3892/or.2015.4398 | No *P. gingivalis* analyses |
| 1. Kinros et al | 2022 | Annals of Oncology | 10.1016/j.annonc.2022.07.496 | Poster/Abstract |
| 1. Kong et al | 2021 | Pathology and Oncology Research | 10.3389/pore.2021.628942  Retracted: 10.3389/pore.2023.1611616 | No healthy controls |
| 1. Kong et al | 2023 | Gut | 10. 1136/ gutjnl- 2022- 327156 | Different outcome |
| 1. Kvich et al | 224 | Gut Microbes | 10.1080/19490976.2024.2350156 | Different outcome |
| 1. Löwenmark et al | 2024 | J Translational Med | 10.1186/s12967-024-05720-8 | Different outcome |
| 1. Li et al | 2023 | Cancer Epidemiol Biomarkers Prev | 10.1158/1055-9965.EPI-22-0724 | Different outcome |
| 1. Liang et al | 2017 | Clinical Cancer Research | 10.1158/1078-0432.CCR-16-1599 | No *P. gingivalis* analyses |
| 1. Liu et al | 2020 | BioMed Research International | 10.1155/2020/2948282 | No *P. gingivalis* analyses |
| 1. Loftus et al | 2021 | BMC Microbiology | 10.1186/s12866-021-02153-x | No original data |
| 1. Lu et al | 2023 | Am J Cancer Res | ISSN:2156-6976/ajcr0153528 | Inappropriate design |
| 1. Marchesi et al | 2011 | PloS One | 10.1371/journal.pone.0020447 | No healthy controls |
| 1. Messaritakis et al | 2024 | Cancers | 0.3390/cancers1610192 | No *P. gingivalis* analyses |
| 1. Mira-Pascual et al | 2015 | Journal of Gastroenterology | 10.1007/s00535-014-0963-x | No *P. gingivalis* analyses |
| 1. Miranda-López et al | 2024 | Letters in Applied Microbiology | 10.1093/lambio/ovae048 | No *P. gingivalis* analyses |
| 1. Mjelle et al | 2019 | BMC Cancer | 10.1186/s12885-019-5330-0 | No *P. gingivalis* analyses. No healthy controls |
| 1. Nakatsu et al | 2015 | Nature Communications | 10.1038/ncomms9727 | No *P. gingivalis* analyses |
| 1. Pignatelli et al | 2021 | Cancers | 10.3390/cancers13051032 | No healthy controls |
| 1. Priselac et al | 2022 | United European Gastroenterology Journal | 10.1002/ueg2.12294 | Poster/Abstract |
| 1. Purcell et al | 2017 | Scientific reports | 10.1038/s41598-017-11237-6 | No healthy controls |
| 1. Rezasoltani et al | 2020 | International journal of molecular sciences | 10.3390/ijms21238968 | Different outcome. |
| 1. Rezasoltani et al | 2018 | Microbial pathogenesis | 10.1016/j.micpath.2018.08.035 | Different outcome. |
| 1. Roesel et al | 2022 | British Journal of Surgery | 10.1093/bjs/znac181.014 | Poster/Abstract |
| 1. Russo et al | 2018 | Frontiers in Microbiology | 10.3389/fmicb.2017.02699 | No *P. gingivalis* analyses |
| 1. Serrano et al | 2021 | Nutrients | 10.3390/nu13020363 | No *P. gingivalis* analyses |
| 1. Shen et al | 2020 | Translational Oncology | 10.1016/j.tranon.2020.100772 | No *P. gingivalis* analyses |
| 1. Senthakumaran et al | 2023 | Eur J Clin Microbiol Infect Dis | 10.1007/s10096-023-04551-7 | No *P. gingivalis* analyses |
| 1. Sinha et al | 2016 | PloS one | 10.1371/journal.pone.0152126 | No *P. gingivalis* analyses |
| 1. Sun et al | 2022 | Frontiers in Oncology | 10.3389/fonc.2022.982744 | No *P. gingivalis* analyses |
| 1. Tarallo et al | 2020 | mSystems | 10.1128/mSystems.00072-20 | No original data |
| 1. Tito et al | 2024 | Nat Med | 10.1038/s41591-024-02963-2 | No *P. gingivalis* analyses |
| 1. Tortora et al | 2024 | Cancer Res | [10.1158/1538-7445.AM2024-6696](https://doi.org/10.1158/1538-7445.AM2024-6696) | Poster/Abstract |
| 1. Voghtmann et al | 2018 | Gastroenterology | 10.1016/S0016-5085(18)32887-7 | Poster/Abstract |
| 1. Wallace et al | 2020 | Cancer Research | 10.1158/1538-7445.MVC2020-B33 | Poster/Abstract |
| 1. Wang et al | 2021 | Frontiers in oncology | 10.3389/fonc.2021.685641 | No *P. gingivalis* analyses |
| 1. Wang et al | 2012 | The ISME Journal | 10.1038/ismej.2011.109 | No *P. gingivalis* analyses |
| 1. Warren et al | 2013 | Microbiome | 10.1186/2049-2618-1-16 | No healthy controls |
| 1. Weir et al | 2013 | PLoS One | 10.1371/journal.pone.0070803 | No *P. gingivalis* analyses |
| 1. Wu et al | 2013 | Microbial Ecology | 10.1007/s00248-013-0245-9 | No *P. gingivalis* analyses |
| 1. Xiong et al | 2022 | Frontiers in Cellular and Infection Microbiology | 10.3389/fcimb.2022.1036946 | No *P. gingivalis* analyses |
| 1. Yachida et al | 2019 | Nature Medicine | 10.1038/s41591-019-0458-7 | No healthy controls |
| 1. Yang et al | 2019 | Experimental and Molecular Medicine | 10.1038/s12276-019-0313-4 | No *P. gingivalis* analyses |
| 1. Yang et al | 2019 | Scientific Reports | 10.1038/s41598-019-45588-z | No *P. gingivalis* analyses |
| 1. Yang et al | 2023 | Frontiers in Oncology | 0.3389/fonc.2023.112722 | No *P. gingivalis* analyses |
| 1. Yazici et al | 2017 | Gut | 10.1136/gutjnl-2016-313321 | No *P. gingivalis* analyses |
| 1. Young et al | 2019 | Journal of Pathology | 10.1002/path.5345 | Poster/Abstract |
| 1. Young et al | 2019 | Journal of Pathology | 10.1002/path.5345 | Poster/Abstract |
| 1. Yu et al | 2017 | Gut | 10.1136/gutjnl-2015-309800 | No *P. gingivalis* analyses |
| 1. Zackular et al | 2014 | Cancer Prevention Research | 10.1158/1940-6207.CAPR-14-0129 | No *P. gingivalis* analyses |
| 1. Zhang et al | 2018 | BMC microbiology | 10.1186/s12866-018-1232-6 | No *P. gingivalis analyses* |
| 1. Zhang et al | 2020 | Theranostics | 10.7150/thno.49515 | No *P. gingivalis* analyses |
| 1. Zhang et al | 2022 | Frontiers in microbiology | 10.3389/fmicb.2022.1005201 | No *P. gingivalis* analyses |
| 1. Zhang et al | 2023 | Frontiers in Microbiology | 10.3389/fmicb.2023.1182346 | Different outcome |
| 1. Zhao et al | 2023 | International Journal of Radiation Oncology | [10.1016/j.ijrobp.2023.06.2442](https://doi.org/10.1016/j.ijrobp.2023.06.2442) | Poster/Abstract |
| 1. Zhou et al | 2022 | Journal of clinical laboratory analysis | 10.1002/jcla.24359 | Inappropriate design: Narrative review |
| 1. Zhou et al | 2022 | FEMS Microbiology Letters | 10.1093/femsle/fnac023 | No original data |
| 1. Zorron et al | 2020 | J.Gastroenterol. Hepatol. | 10.1111/jgh.14868 | No healthy controls |
| 1. Zwinsová et al | 2021 | Cancers | 10.3390/cancers13194799 | No healthy controls |

Supplementary Table S3: Grading of Recommendations Assessment, Development and Evaluation (GRADE) assessment

| **GRADE criteria** | **Rating** (&) | **Footnotes**  **(**explain reasons for down- or upgrading**)** | **Quality of the evidence**  (Mark one) |
| --- | --- | --- | --- |
| **Outcome: Colorectal cancer** | | | |
| **Study design** | **Observational studies**  (it starts as low quality) |  | ⊕⊕⊕⊕ High  ⊕⊕⊕ Moderate  ⊕⊕ Low  **⊕ Very Low** |
| **Study limitations (Risk of bias)** (a) | Very serious (-2) | Problems with the representativeness of cases, definition of controls, non-response rate, quality of laboratory analyses and blind analyses. |  |
| **Inconsistency** (b) | Very serious (-2) | It was not possible to perform a heterogeneity analysis due to the scarcity number of studies and lack of information provided. |  |
| **Indirectness** (c) | Very serious (-2) | In the absence of well-designed prospective cohort studies, evidence was based on case-control studies where samples were obtained at the same time of confirmed diagnoses. |  |
| **Imprecision** (d) | Very Serious (-2) | A total number of participants over 400 was evaluated in 20 % of studies. |  |
| **Publication bias** (e) | Strongly suspected (-1) | Most results come from small studies and no proper analyses of publication could be performed. |  |
| **Other** (f)  (upgrading factors, circle all that apply) | Large effect (0)  Dose response (Not evaluated)  No plausible confounding (0) | No effect size could be calculated, the dose response effect is not possible to be evaluated and there are plausible confounding affecting the effect (see Table 3 and 4) |  |

(a) Study limitations (Risk of bias): based on Newcastle-Otawa Scale for case-control studies (see table 2 and Figure 2), quality controls and blind analysis reported.

(b) Inconsistency: based on the evaluation of heterogeneity (*Q* statistic and I^2^ statistic) in this meta-analysis.

(c) Indirectness: refers to how well the evidence included in the review answers the review question related to population, intervention, comparator or outcome used in included studies.

(d) Imprecision: results are considered imprecise when studies include only relatively few participants (a total number of participants is less than 400 for continuous outcome information is considered to be insufficient).

(e) Publication bias: as evaluated in this meta-analysis.

(f) Other: the three major possible reasons to upgrade the quality of evidence are a large magnitude of effect, a dose-response relationship and the effect of all plausible confounding factors.

(&) Rating for (a), (b), (c) and (d): Not affected, No serious (-1) or Very serious (-2); Rating for (e): Undetected or Strongly suspected (-1); Rating for any of the three categories in Other (f): 0, +1 or +2.
